# Associations between adverse childhood experiences and health outcomes in adults aged 18–59 years

**DOI:** 10.1371/journal.pone.0211850

**Published:** 2019-02-07

**Authors:** Xuening Chang, Xueyan Jiang, Tamara Mkandarwire, Min Shen

**Affiliations:** Department of Maternal and Child Health, School of Public Health, Tongji Medical College, Huazhong University of Science and Technology, Wuhan, Hubei, China; Stellenbosch University, SOUTH AFRICA

## Abstract

**Background:**

Adverse childhood experiences (ACEs) have been associated with poor health status later in life. The objective of the present study was to examine the relationship between ACEs and health-related behaviors, chronic diseases, and mental health in adults.

**Methods:**

A cross-sectional study was performed with 1501 residents of Macheng, China. The ACE International Questionnaire (ACE-IQ) was used to assess ACEs, including psychological, physical, and sexual forms of abuse, as well as household dysfunction. The main outcome variables were lifetime drinking status, lifetime smoking status, chronic diseases, depression, and posttraumatic stress disorder. Multiple logistic regression models were used to examine the associations between overall ACE score and individual ACE component scores and risk behaviors/comorbidities in adulthood after controlling for potential confounders.

**Results:**

A total of 66.2% of participants reported at least one ACE, and 5.93% reported four or more ACEs. Increased ACE scores were associated with increased risks of drinking (adjusted odds ratio [AOR] = 1.09, 95% confidence intervals [CI]: 1.00–1.09), chronic disease (AOR = 1.17, 95% CI: 1.06–1.28), depression (AOR = 1.37, 95% CI: 1.27–1.48), and posttraumatic stress disorder (AOR = 1.32, 95% CI: 1.23–1.42) in adulthood. After adjusting for confounding factors, the individual ACE components had different impacts on risk behavior and health, particularly on poor mental health outcomes in adulthood.

**Conclusions:**

ACEs during childhood were significantly associated with risk behaviors and poor health outcomes in adulthood, and different ACE components had different long-term effects on health outcomes in adulthood.

## Introduction

Adverse childhood experiences (ACEs) are potentially traumatic events that can have negative and persistent effects on health later in life [1–3]. ACEs include psychological, physical, and sexual forms of abuse, as well as household dysfunction, such as substance abuse, mental illness, and violence [1]. Previous studies have shown that ACEs are associated with premature death [4, 5], risk behaviors [6], increased comorbid conditions, and chronic diseases [7, 8] such as cancer [2, 9] in adulthood.

The accumulating evidence for the negative impact of ACEs on health outcomes in adulthood mean that they are now considered a public health concern [10]. ACEs have a dose–response relationship with many health outcomes, including heart rate responses to stress and chronic health conditions such as coronary heart disease and stroke [3, 11, 12]. In addition, ACEs are significantly associated with depression [13, 14], insufficient sleep [15], and diabetes [16]. A systematic review found significant associations between ACE scores and cancer incidence in adulthood [2]. Similarly, population-based Swedish cohort studies have longitudinally linked measurable indicators of childhood household dysfunction with poor mental health outcomes and psychotropic medication use [17]. The 1958 British birth cohort (National Child Development Study) found a link between ACEs and cancer risk in adulthood as well as premature all-cause mortality [4].

Despite this large body of research, it is not clear whether findings from previous ACE-related studies can be considered representative, as they have primarily been conducted in developed countries [18, 19]. Although ACEs are more prevalent in low- and middle-income countries owing to limited resources and less social health care [7, 20], little is known about their health impacts. For example, there is limited information on the differential effects of individual ACE components on health-related risk behavior and health outcomes in adulthood in underrepresented populations.

The primary objective of the current study was to examine the relationships between individual ACE components and high-risk health behaviors in adulthood in China. In addition, we examined whether the long-term health outcomes associated with ACEs in China were similar to those in other parts of the world.

## Materials and methods

### Study sample

A cross-sectional study was carried out in Macheng city, Hubei province, China, in May 2014. The baseline sample was adults aged 18–59 years from 13 collective rural communities. The participants were selected from three communities (Drum Tower Community, Longchi Community, and South Lake Community) using random cluster sampling. First, 13 communities were listed according to their distance from the main county urban area. Second, we randomly selected three communities near the downtown areas to take part in the survey. Finally, adults aged 18–59 years in the selected communities were invited to participate during the survey period. The survey was conducted by trained investigators and facilitated by staff of Macheng Center for Disease Prevention and Control. Participants completed an anonymous, self-administered questionnaire. Trained investigators from the research team guided respondents through the process of survey completion. All participants agreed to participate and signed a written informed consent form. The study was approved by the institutional ethical review board of Tongji Medical College, Huazhong University of Science and Technology, China. According to previous study the prevalence rate of depression was 21–57% among people suffered from adverse childhood events (ACE) [1], we supposed p = 23%, the sample size was calculated by the formula: *n* = 400×[(1-*p*)/*p*] = 1339.

### Measures

Participants’ demographic characteristics were recorded using questionnaires, and included age, sex, residence, whether they were the only child during their childhood, number of years of education, occupation, body mass index (BMI), and monthly income. Sex (male/female), residence (urban/rural), only-child status (yes/no), and occupational status (employed/unemployed) were dichotomized. The number of years of education was categorized as ≤6, 7–9, 10–11, and ≥12 years. Monthly income was categorized as <1000, 1000–1999, 2000–2999, and ≥3000 yuan (RMB).

ACEs were assessed using the Adverse Childhood Experiences International Questionnaire (ACE-IQ) developed by the World Health Organization [21]. The ACE-IQ questionnaire has good reliability and validity in China [22, 23]. It includes four subcategories: neglect, abuse, violence, and family dysfunction. Experiences are further classified as emotional neglect, physical neglect, emotional abuse, physical abuse, sexual abuse, family dysfunction, peer violence, witnessing community violence, and exposure to collective violence. Each respondent was asked if they had experienced various adverse events during their childhood (prior to the age of 18 years). ACE scores were recorded as total counts for the number of questions to which respondents answered that they had experienced an ACE. To investigate the influence of specific types of ACEs, questions were categorized according to neglect (physical or emotional), abuse (physical, emotional, or sexual), family dysfunction (parents separated/divorced, family drug abuse, family mental disorders, or family crime), and violence (being bullied, domestic violence, witness of violence, or collective violence).

Alcohol drinking behavior was assessed by questions on alcohol consumption status. Drinking behavior was defined as drinking alcohol (liquor, beer, or wine) at least once per month. Participants were asked, “What is your alcohol drinking status?”, to which they could respond as follows: 1) Current drinker, 2) Have never drunk alcohol, or 3) Former drinker. For current drinkers, we further asked, “How many times have you drunk alcohol in the past year?”, to which they could respond as follows: 1) At least 3 times/week, 2) 1–2 times/week, 3) 1–2 times/month, or 4) less than once/month. Smoking behavior was defined as smoking more than 100 cigarettes over at least 3 months during their lifetime. Smoking status was classified as current smoker, former smoker, and never smoked. BMI was calculated as weight in kilograms divided by the square of height in meters. Chronic disease was determined based on participants’ hospital medical records, and was defined as “positive” if participants reported that they had at least one kind of chronic disease, including hypertension, diabetes, coronary heart disease, respiratory diseases, cerebrovascular disease (stroke), digestive system diseases, and urinary system diseases.

The Center for Epidemiological Studies Depression Scale (CES-D) was used to assess depression [24]. The CES-D scale consists of 20 self-reported items; it was designed to identify populations at risk of developing depression, and is widely used to screen for probable clinical depression and to measure the severity of depression symptoms. Respondents were asked to indicate whether they had experienced the symptoms described during the past week (not at all, rarely, sometimes, and often). The total score was the summed score of all items. This study used the Chinese version of the CES-D, on which a score equal to or greater than 20 indicates depression [25]. Posttraumatic stress disorder (PTSD) was assessed using the PTSD Checklist-Civilian Version (PCL-C) [26], which is a 17-item self-reported measure of PTSD symptoms. The frequency of symptom occurrence during the past month was rated on a 5-point scale, ranging from 1–5, and the total score was computed by summing scores for all items. This study used the Chinese version of the scale, on which a score equal to or greater than 50 indicates PTSD. The PCL-C has shown good test–retest reliability (r = 0.66) and excellent internal consistency (α = 0.92) in the Chinese population [27].

### Statistical analysis

Differences in demographic characteristics between the four ACE score categories were analyzed using chi-square tests and t-tests, according to variable type. The t-test was used to analyze differences in average ACE score by risky behaviors and comorbidities in adulthood. Separate multiple logistic regression models were used to assess the associations between ACE score (the independent variable) and health-related risk behaviors (i.e., drinking and smoking) and comorbidities in adulthood (the dependent variables), while controlling for confounding factors. Potential confounding factors were age, sex, BMI, residence, only-child status, years of education, occupation, and monthly income. Finally, multiple logistic regression was used to assess the associations between ACE components and health-related risk behaviors and comorbidities in adulthood, while adjusting for confounders. Separate models were run with each health-related risky behavior and comorbidity as the dependent variables; ACE components were the primary independent variables, and age, sex, BMI, residence, education, only-child status, occupation, and income were confounding variables. The odds ratios (OR) and 95% confidence intervals (CI) were estimated from the multiple logistic regression models. SAS version 9.4 (SAS Institute Inc., Cary, NC, USA) was used for all statistical analyses. A two-sided *P*-value of <0.05 indicated statistical significance.

## Results

Among the 1767 eligible participants, 1501 (84.9%) returned a completed ACE questionnaire. Of these, 66.2% reported having experienced at least one ACE and 5.93% reported experiencing four or more ACEs. Table 1 shows the prevalence of ACE categories by demographic characteristics. Childhood abuse, family dysfunction, and violence were significantly more prevalent in men (45.92%, 36.87%%, and 28.70%) than in women (27.48%, 27.39%, and 18.99%, *P* < 0.001), respectively.

**Table 1:** Demographics of participants aged 18–59 years by ACE categories.

Characteristics	N	Abuse		Neglect		Family dysfunction		Violence	
		n (%)	*P*	n (%)	*P*	n (%)	P	n (%)	*P*
Age, years	1501	36.23±9.23	.270	37.25±9.53	< .001	38.36±9.14	< .001	36.16±9.60	.554
Gender									
Male	453	208(45.92)	< .001	163(35.98)	.05	167(36.87)	< .001	130(28.70)	< .001
Female	1048	288(27.48)		433(41.32)		287(27.39)		199(18.99)	
Residence									
Rural	750	205(27.33)	< .001	301(40.13)	.74	206(27.47)	.02	161(21.47)	.67
Urban	751	291(38.75)		295(39.28)		248(33.02)		168(22.37)	
Only child									
Yes	179	52(29.05)	.23	78(43.58)	.26	42(23.46)	.04	34(18.99)	.31
No	1322	444(33.59)		518(39.18)		412(31.16)		295(22.31)	
Years of education, year									
≤6	94	24(25.53)	.003	25(26.60)	.02	27(28.72)	.77	14(14.89)	.12
7–9	505	157(31.09)		214(42.38)		145(28.71)		125(24.75)	
10–11	401	119(29.68)		169(42.14)		126(31.42)		81(20.20)	
≥12	501	196(39.12)		188(37.52)		156(31.14)		109(21.76)	
Occupation									
Employed	1281	420(32.79)	.61	503(39.27)	.40	392(30.60)	.47	265(20.69)	.005
Unemployed	220	76(34.55)		93(42.27)		62(28.18)		64(29.09)	
BMI	1501	21.99±2.96	.092	22.07±2.97	.004	22.14±3.11	.012	21.86±3.14	.725
Income/month, Yuan									
<1000	285	91(31.93)	.19	130(45.61)	.06	86(30.18)	.35	79(27.72)	.01
1000–1999	569	172(30.23)		220(38.66)		158(27.77)		103(18.10)	
2000–2999	464	167(35.99)		185(39.87)		149(32.11)		104(22.41)	
≥3000	183	66(36.07)		61(33.33)		61(33.33)		43(23.50)	

Table 2 shows the average ACE scores by risky behaviors and health-related outcomes. Significantly higher average ACE scores were found for participants with current-drinking, current smoking, chronic diseases, depression, and PTSD compared with their counterparts (*P* < 0.001). A dose–response effect was associated with more risk behaviors, depression, and PTSD as ACE scores increased. Table 3 shows the multivariate logistic regression results of the associations between ACE score and health-related risk behaviors and morbidities. Generally, the adjusted OR (AOR) for health-related risk behaviors and morbidities increased as ACE scores increased. Higher ACE scores were associated with higher risk of being lifetime drinkers (AOR = 1.09, 95% CI: 1.00–1.19), chronic diseases (AOR = 1.17, 95% CI: 1.06–1.28), depression (AOR = 1.37, 95% CI: 1.27–1.48), and PTSD (AOR = 1.32, 95% CI: 1.23–1.42) in adulthood.

**Table 2:** Average ACE scores compared between groups with and without health related risky behaviors and health outcomes.

Risky behaviors and health outcomes			ACE Score		
			Mean ± SD	*t* value	*P* value
Drinking-lifetime	Yes	480	1.94 ± 1.73	4.995	0.000
	No	1021	1.49 ± 1.52		
Smoking-current	Yes	256	2.17 ± 1.76	5.189	0.000
	No	1245	1.52 ± 1.52		
Chronic diseases	Yes	196	2.15 ± 1.83	4.224	0.000
	No	1305	1.56 ± 1.55		
Depression	Yes	319	2.28 ± 1.86	7.199	0.000
	No	1182	1.46 ± 1.47		
PTSD	Yes	469	2.16 ± 1.72	8.150	0.000
	No	1032	1.40 ± 1.48		

**Table 3:** Multivariate logistic regression models for relationship between ACE score and risky behaviors and health outcomes.

Risky behaviors and health outcomes	ACE Score	
	AOR (95% CI)	*P* value
Drinking-lifetime	1.09 (1.00, 1.19)	0.043
Smoking-current	1.09 (0.98, 1.21)	0.113
Chronic diseases	1.17 (1.06, 1.28)	0.001
Depression	1.37 (1.27, 1.48)	0.000
PTSD	1.32 (1.23, 1.42)	0.000

Note: Adjusting for age, gender, BMI, residence, only-child, years of education, occupation, and monthly income.

The associations between ACE components and health outcomes are shown in Tables 4 and 5. Current-drinkers were more likely to report experiencing domestic violence (AOR = 1.68, 95% CI: 1.13–2.50) during childhood than non-drinkers. Current-smokers were more likely to report experiencing physical abuse (AOR = 1.66, 95% CI: 1.10–2.48, *P* < 0.05), physical neglect (AOR = 2.04, 95% CI: 1.28–3.26) and sexual abuse (AOR = 2.78, 95% CI: 1.26–6.12) during childhood than non-smokers. The presence of chronic diseases was significantly associated with childhood emotional abuse (AOR = 1.65, 95% CI: 1.18–2.31, *P* < 0.01) and physical abuse (AOR = 1.81, 95% CI: 1.28–2.56, *P* < 0.01), being bullied (AOR = 2.82, 95% CI: 1.40–5.65, *P* < 0.01), and family drug abuse (AOR = 3.40, 95% CI: 1.38–8.36, *P* < 0.01). The individual ACE components had different effects on depression in adulthood. Adulthood PTSD was significantly associated with sexual abuse (AOR = 2.84, 95% CI: 1.71–4.73), domestic violence (AOR = 2.52, 95% CI: 1.83–3.45), community violence (AOR = 1.45, 95% CI: 1.07–1.96), and emotional and physical abuse during childhood.

**Table 4:** Multivariate logistic regression models for relationship between ACE categories and risky behaviors and health outcomes.

Risky behaviors and health outcome	Emotional neglect	Emotional abuse	Physical abuse	Physical neglect	Sexual abuse	Domestic violence	Witness of community violence	Collective violence
Drinking-current	1.04 (0.77,1.39)	1.23 (0.90,1.69)	1.17 (0.84,1.62)	1.04 (0.73,1.48)	0.85 (0.45,1.63)	1.68* (1.13,2.50)	1.20 (0.82,1.76)	1.18 (0.43,3.23)
Smoking-current	0.95 (0.64,1.41)	1.03 (0.68,1.54)	1.66* (1.10,2.48)	2.04** (1.28,3.26)	2.78* (1.26,6.12)	1.25 (0.77,2.04)	1.53 (0.95,2.46)	0.96 (0.25,3.75)
Chronic diseases	0.87 (0.62,1.22)	1.65** (1.18,2.31)	1.81** (1.28,2.56)	0.98 (0.66,1.46)	0.77 (0.36,1.65)	1.48 (0.97,2.26)	1.77** (1.19,2.64)	1.39 (0.50,3.82)
Depression	1.76*** (1.35,2.39)	1.98*** (1.50,2.63)	1.37* (1.02,1.86)	1.99*** (1.48,2.70)	3.05*** (1.82,5.12)	1.80** (1.26,2.56)	1.95*** (1.40,2.70)	1.38 (0.60,3.19)
PTSD	1.32* (1.04,1.67)	1.92*** (1.49,2.47)	1.33* (1.01,1.73)	1.82*** (1.38,2.38)	2.84*** (1.71,4.73)	2.52*** (1.83,3.45)	1.45* (1.07,1.96)	1.27 (0.60,2.72)

Note: Boldface indicates statistical significance (*P<0.05; **P<0.01; ***P<0.001)

**Table 5:** Multivariate logistic regression models for relationship between ACE categories and risky behaviors and health outcomes.

Risky behaviors and health outcomes	Being bullied	Parents separated /divorced	Family drug abuse	Family mental disorder	Family crime
Drinking-lifetime	0.87 (0.41, 1.86)	1.06 (0.76,1.49)	2.07 (0.77,5.55)	2.78 (0.91,1.11)	1.28 (0.33,4.88)
Smoking-current	0.53 (0.21,1.39)	1.24 (0.79,1.95)	0.65 (0.16,2.68)	0.39 (0.04,3.66)	1.09 (0.26,4.65)
Chronic diseases	2.82** (1.40, 5.65)	1.08 (0.75,1.55)	3.40** (1.38,8.36)	1.45 (0.45,4.68)	3.30 (0.82,13.30)
Depression	2.62** (1.45, 4.75)	1.40* (1.04,1.89)	0.45 (0.13,1.55)	3.91** (1.46,10.47)	0.62 (0.13,2.97)
PTSD	1.63 (0.91, 2.91)	1.26 (0.97,1.65)	1.29 (0.56,3.01)	2.51 (0.95,6.62)	1.78 (0.53,5.98)

Note: Boldface indicates statistical significance (*P<0.05; **P<0.01; ***P<0.001)

## Discussion

This study confirmed that ACEs were associated with higher odds of risky behavior, chronic disease, and mental disorders in adulthood after controlling for relevant demographic and socioeconomic factors. Our results support the hypothesized dose–response effect of increased exposure to ACEs during childhood on the presence of risk behaviors and morbidity in adulthood. The more ACEs in childhood, the higher the likelihood of risky behaviors and morbidity in adulthood, a finding consistent with previous studies [12,20,[28–30]. More importantly, this study examined the cumulative effects of ACEs on health outcomes in adulthood and also evaluated the differential health impacts of each ACE component. The five ACE categories (emotional abuse, witnessing community violence, domestic violence, collective violence, and family drug abuse) had significantly different effects on depression. Previous studies have reported similar associations [3, 31].

The overall prevalence of at least one ACE in this Chinese sample was 47% to 82%, which is similar to the prevalence reported in studies from other developed and developing countries [7, 32–34]. Our results are also in accordance with previous research that has suggested that ACEs have a dose–response relationship in increasing risky behaviors and morbidity in adulthood [12,20]. Frankenberger and colleagues [35] reported that experiencing more ACEs was positively associated with alcohol use during pregnancy. One cohort study showed that the OR for more than three ACEs and risk of psychotropic medication was 2.4 (95% CI: 2.3–2.5) for young women and 3.1 (95% CI: 2.9–3.2) for young men [36]. In the present study, participants who reported ≥4 ACEs had 1.83-fold odds of increased lifetime smoking, 5.4-fold odds of increased depression, 1.76-fold odds of developing chronic diseases, and 4.01-fold odds of PTSD in adulthood, compared with those who reported no ACEs.

We also evaluated the differential impacts of individual ACE categories on risk behaviors and morbidity in adulthood. To our knowledge, this is the first study on ACEs and their impact on adult health in China. We found, for example, that individuals who reported experiencing emotional abuse during childhood had an increased risk of depression (by 1.98-fold) and PTSD (by 1.92-fold) in adulthood compared with those who did not suffer from emotional abuse. Physical abuse during childhood was significantly related to adult smoking, chronic disease, and poor mental health (ORs ranged from 1.33 to 1.81). Domestic violence, community violence, and sexual abuse during childhood were significantly associated with an increased risk of PTSD in adulthood (ORs ranged from 1.45 to 2.84), which is consistent with previous studies [37, 38]. Abuse and neglect have a deleterious impact on the emergent attachment system and can lead to emotional dysregulation and an increase in cortisol levels [39]. Such ongoing arousal and reactive responses can be considered symptoms of depression and PTSD [40, 41]. Additionally, participants reporting chronic diseases in adulthood were more likely to have experienced physical abuse (1.81-fold), being bullied (2.82-fold), and family drug abuse (3.40-fold) during childhood. These negative outcomes are largely a result of the influence of physiological reactions to psychosocial stress (such as toxic stress and allostatic load), which can cumulate and interact, leading to damage to the metabolic, cardiovascular, immune, and nervous systems [39]. This early damage could predispose a child to a series of health challenges in later life and result in chronic diseases. We found that physical neglect and abuse was associated with a higher risk of mental health problems in later life. Prospective studies are warranted to clarify the mechanisms driving these associations.

## Strengths and limitations of this study

This study assessed a wide range of ACEs using the ACE-IQ and described ACE-related factors in China, which is a middle-income country; our results therefore shed light on this issue in a different socioeconomic and demographic context than most previous studies, which have been carried out in high-income countries. Our study supports previous findings on the cumulative impacts of ACEs on risk behavior, chronic diseases, depression, and PTSD in adulthood.

This study has several limitations. First, this was a cross-sectional study: ACE experiences were retrospectively recalled in adulthood and so may have been subject to recall bias; it is difficult to determine how much self-reported ACEs reflect the “true” situation. The cross-sectional design of the study also precluded inference of causality. Second, although this study measured variables deemed important in the literature, our data set did not permit measurement of all potential confounders, such as adult adverse events. Finally, the data cannot be generalized to the entire Chinese population because the sample was drawn from one city in central China.

## Conclusions

In summary, this study demonstrates the long-term effects of exposure to ACEs and the differential influences of ACE type on risk behavior and chronic diseases in adulthood. Men and lower-income participants had a higher prevalence of ACEs than women and higher-income participants, respectively. As the ACE score increased, the risky odds for PTSD, chronic disease, depression, and smoking and drinking behavior during adulthood significantly increased. Experience of violent ACEs was associated with depression and PTSD in adulthood. Our results therefore highlight the serious long-term consequences of ACEs. These findings may facilitate better identification of individuals at risk and the development of effective interventions to protect children from abuse and violence in China.
